# Focused Cardiac Ultrasound and Doppler Hemodynamics in Nephrology Practice

**DOI:** 10.3390/diagnostics16213543

**Published:** 2026-10-28

**Authors:** Abhilash Koratala, Nathaniel Reisinger, Aala Jaberi, Stephanie Torres Rodriguez, Amir Kazory

**Affiliations:** 1 HUB Room A7333, Divisions of Nephrology and Surgical Critical Care, Medical College of Wisconsin, 8701 W Watertown Plank Rd, Milwaukee, WI 53226, USA; 2 Renal-Electrolyte and Hypertension Division, University of Pennsylvania, Philadelphia, PA 19104, USA; 3 Saint Louis Kidney Consultants, St. Louis, MO 63141, USA; 4 Section of Nephrology, Boston University Medical Center, Boston, MA 02118, USA; aala.jaberi@bmc.org; 5 Division of Nephrology, Department of Internal Medicine, University of Texas Southwestern Medical Center, Dallas, TX 75390, USA; stephanie.torresrodriguez@utsouthwestern.edu; 6 Division of Nephrology, Hypertension and Renal Transplantation, University of Florida, Gainesville, FL 32611, USA

**Keywords:** point-of-care ultrasound, echocardiography, nephrology, congestion, Doppler

## Abstract

Point-of-care ultrasonography (POCUS) in nephrology is evolving beyond kidney imaging and procedural guidance toward integrated hemodynamic assessment. Distinguishing reduced forward flow from elevated filling pressures and venous congestion remains challenging in patients with acute kidney injury and cardiorenal syndromes. This educational review discusses the clinical reasoning behind incorporating basic and advanced cardiac POCUS into nephrology practice. We highlight the interpretation and limitations of ventricular size and function, inferior vena cava assessment, stroke volume and left ventricular outflow tract Doppler, left ventricular filling pressures, right-sided and pulmonary artery pressures, and right ventricular outflow tract Doppler, with emphasis on how these findings may influence fluid removal, volume administration, and vasoactive therapy. The complementary role of venous excess ultrasound (VExUS) in assessing the downstream consequences of systemic venous congestion is also discussed. Rather than relying on isolated sonographic parameters or rigid cutoffs, cardiac POCUS findings should be integrated with multisystem ultrasound and the broader clinical context. Familiarity with these principles can help nephrologists obtain focused, repeatable hemodynamic information at the bedside and translate sonographic findings into more physiologically informed clinical decisions.

## 1. Background

Until recently, nephrology-related point-of-care ultrasonography (POCUS) was largely confined to procedural applications, such as vascular access guidance for dialysis catheter placement, and, at select institutions, diagnostic kidney ultrasonography. Over the past decade, however, there has been growing interest in diagnostic POCUS applications extending beyond the kidneys, paralleling the broader expansion of ultrasonography across medical education and multiple clinical specialties. Increasingly, incoming nephrology trainees are exposed to bedside ultrasonography during medical school and residency and are seeking more comprehensive nephrology-specific POCUS training. Accordingly, the scope of nephrologist-performed POCUS is evolving beyond isolated organ assessment [1]. This broader approach is particularly relevant because cardiovascular hemodynamics influence kidney function across a wide spectrum of kidney diseases and are central to the supportive management of acute kidney injury (AKI) once obstructive nephropathy has been excluded. Kidney function may be adversely affected not only by reduced forward flow, but also by elevated cardiac filling pressures and systemic venous congestion. Consequently, nephrologists benefit from familiarity with cardiac ultrasound applications that provide clinically meaningful information at the bedside.

Growing enthusiasm for advanced Doppler-based applications such as venous excess ultrasound (VExUS) has further expanded the scope of nephrology POCUS [2]. Although these techniques can provide valuable insights into venous congestion and fluid tolerance, their interpretation without adequate cardiac context may be misleading [3]. More broadly, isolated interpretation of VExUS, inferior vena cava (IVC), or lung ultrasound findings may lead to oversimplified or erroneous conclusions. In this educational review, we therefore discuss selected cardiac POCUS applications, emphasizing the clinical reasoning that underpins their day-to-day use.

## 2. Basic Cardiac POCUS

**Figure 1:**
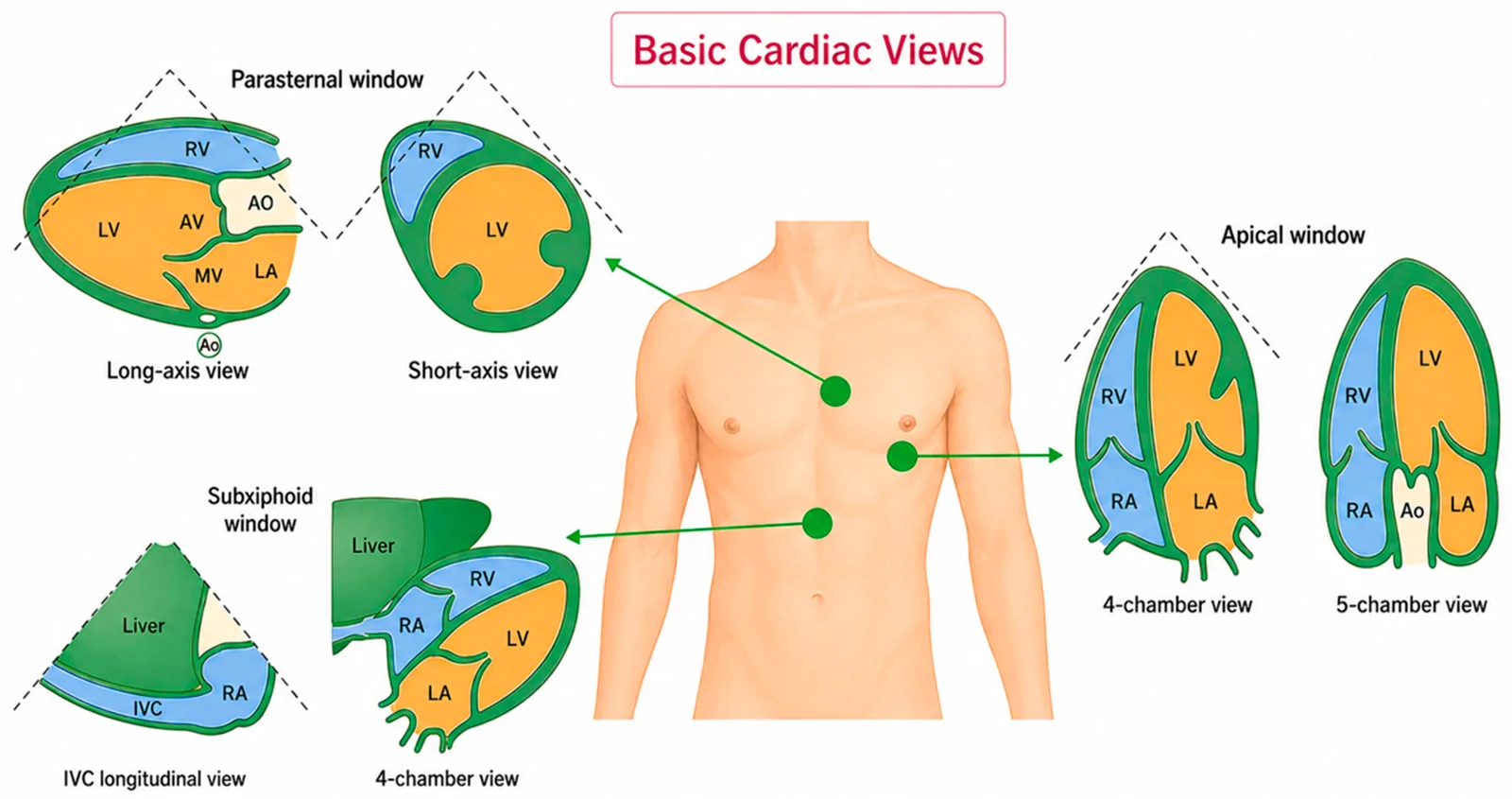
Basic cardiac POCUS views. Schematic illustration of the standard acoustic windows and views used for basic cardiac POCUS, including parasternal long- and short-axis, apical four- and five-chamber, subxiphoid four-chamber, and longitudinal inferior vena cava views. Abbreviations: Ao, aorta; AV, aortic valve; IVC, inferior vena cava; LA, left atrium; LV, left ventricle; MV, mitral valve; RA, right atrium; RV, right ventricle.

Basic cardiac POCUS involves using a limited set of cardiac ultrasound views, primarily with grayscale imaging and color Doppler, to answer focused clinical questions without spectral Doppler techniques [1] (Figure 1). Spectral Doppler, including pulsed-wave and continuous-wave Doppler, provides qualitative and quantitative assessment of blood flow velocities and pressure gradients. It has long been recognized that non-cardiologist physicians can acquire competency in selected basic cardiac POCUS applications with good diagnostic accuracy after relatively short training periods, often outperforming traditional cardiac auscultation for several clinically relevant conditions [4,5,6].

In nephrology, basic cardiac POCUS can rapidly identify or exclude important abnormalities with direct therapeutic implications. For example, a focused examination can be used to detect pericardial effusion and chamber collapse or other sonographic features of cardiac tamponade. POCUS is highly sensitive for detecting even small pericardial effusions and may identify tamponade physiology before hemodynamic compromise or classic clinical findings become apparent [7]. This is particularly relevant in outpatient hemodialysis patients presenting with intradialytic hypotension suspected to be related to pericardial effusion. While obtaining a comprehensive echocardiogram may be complicated by scheduling around thrice-weekly dialysis and resource availability, a bedside examination performed by the treating nephrologist can provide clinically actionable information within minutes and facilitate timely management decisions.

Similarly, left ventricular systolic function can be qualitatively assessed using grayscale imaging by evaluating myocardial thickening, endocardial excursion, and mitral valve motion during diastole. Chamber size assessment also provides valuable hemodynamic information. Right ventricular (RV) dilation may suggest chronic pressure overload, such as pulmonary hypertension, or volume overload states including severe tricuspid regurgitation (TR) or excessive fluid administration [8]. Acute RV dilation should raise suspicion for pulmonary embolism in the appropriate clinical context. Left atrial enlargement may indicate chronically elevated left ventricular filling pressures and diastolic dysfunction, whereas left ventricular dilation in conjunction with reduced systolic function suggests dilated cardiomyopathy. In addition, RV systolic function is commonly assessed by evaluating tricuspid annular motion (tricuspid annular plane systolic excursion [TAPSE]), either qualitatively by visual estimation or quantitatively using M-mode echocardiography.

**Figure 2:**
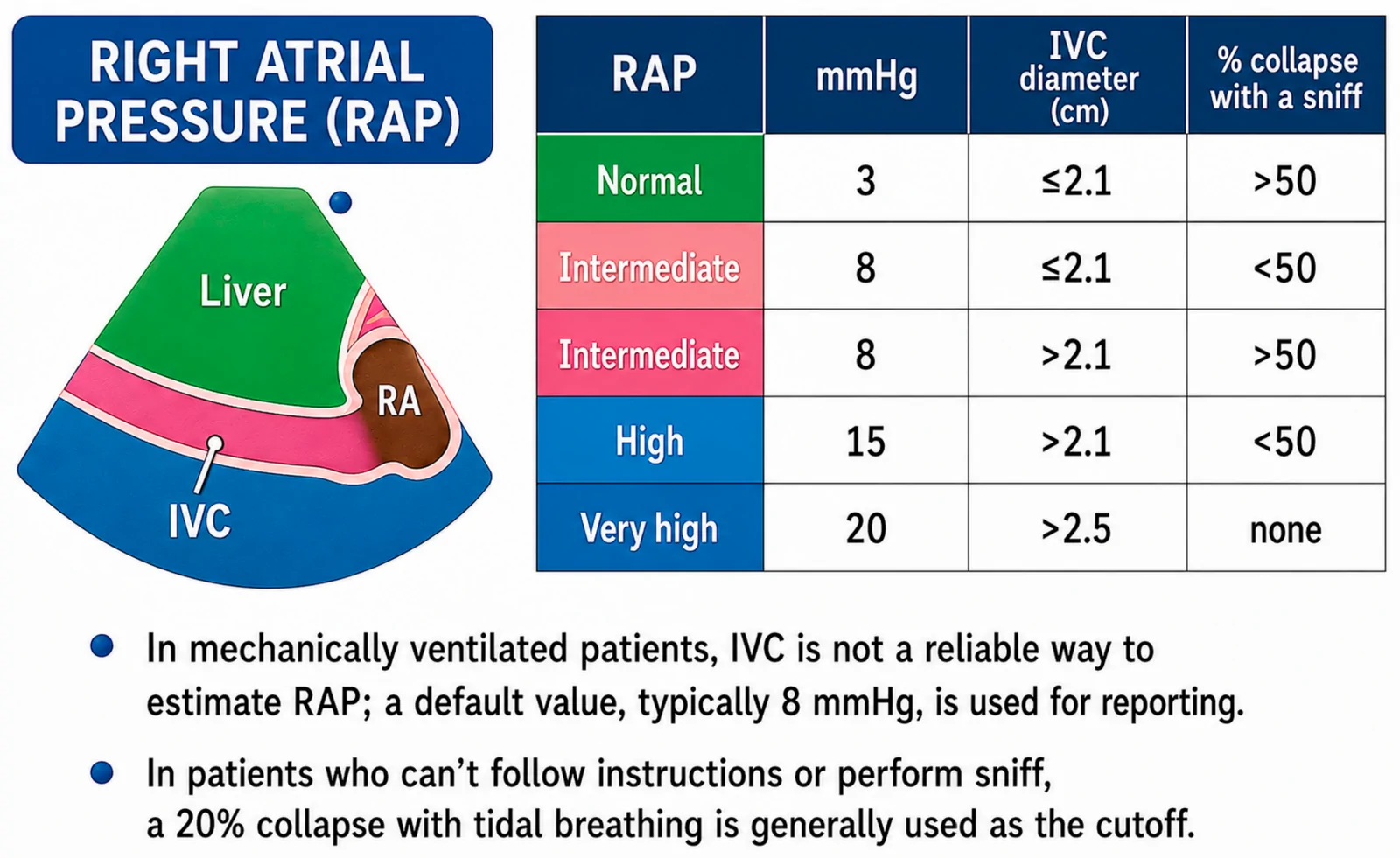
Inferior vena cava assessment for estimation of right atrial pressure based on the current guidelines. IVC diameter and collapsibility during a sniff maneuver are used to estimate RAP in spontaneously breathing patients. The table summarizes commonly used IVC diameter and sniff-induced collapsibility criteria and their corresponding RAP estimates.

**Figure 3:**
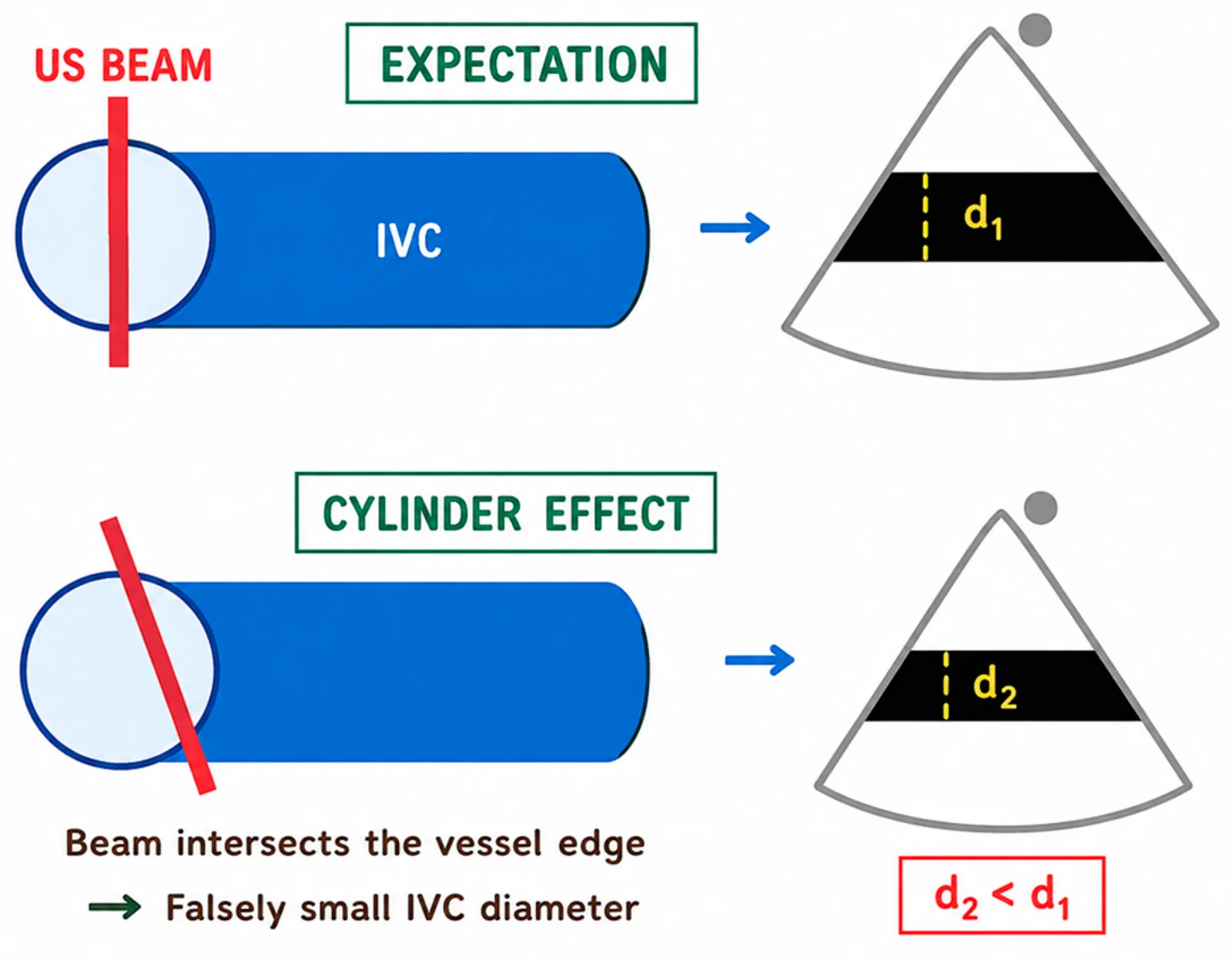
Cylinder effect during inferior vena cava (IVC) assessment. Off-axis insonation intersecting the edge of the IVC may produce a falsely small diameter (d_2_ < d_1_). The cylinder effect can be recognized and avoided by confirming the vessel in the transverse (short-axis) plane. However, diameter should not be measured from an isolated transverse view because of uncertainty regarding the measurement site relative to the right atrium and hepatic vein confluence.

Basic cardiac POCUS also includes IVC assessment. IVC size and respiratory variation provide a noninvasive estimate of right atrial pressure (RAP), with commonly used criteria summarized in Figure 2 [9]. However, it is important to recognize that IVC findings should not be equated directly with “volume status”. A dilated IVC primarily indicates elevated RAP, which may result from diverse pathophysiologic processes including pulmonary hypertension, pericardial disease, or RV dysfunction, in addition to hypervolemia [10]. Consequently, indiscriminate fluid removal in conditions such as severe pulmonary hypertension or pericardial effusion may worsen forward flow and organ perfusion [11]. In addition, mechanically ventilated patients commonly exhibit a dilated and poorly collapsible IVC; therefore, IVC should not be used in isolation to estimate RAP in such settings [9]. Conversely, a small, collapsible IVC simply suggests that RAP is not elevated and, by itself, does not reliably distinguish hypovolemia from euvolemia or high-output states. For example, patients with cirrhosis often have a hyperdynamic circulation and may remain vasodilated with low RAP despite intravascular volume expansion; in such cases, vasoconstrictor therapy may be more appropriate than further fluid administration. Furthermore, elevated intra-abdominal pressure can externally compress the IVC, resulting in a narrowed, “slit-like” or flattened appearance even if the RAP is elevated. This compression may also reduce venous return and cardiac preload, contributing to the decline in cardiac output observed with intra-abdominal hypertension [12]. In such scenarios, internal jugular vein POCUS may provide a complementary assessment of RAP because it evaluates an extra-abdominal vessel. A discrepancy between jugular and IVC findings may support external IVC compression by elevated intra-abdominal pressure and favor measures to relieve the underlying pressure rather than additional volume administration [13]. Both IVC and internal jugular vein ultrasound have important technical limitations in addition to the conceptual considerations discussed above. The IVC is susceptible to the cylinder effect (Figure 3), whereby changes in imaging plane can produce apparent differences in vessel diameter, potentially leading to inconsistent measurements between operators or serial examinations. Respiratory craniocaudal or lateral translation of the IVC may similarly introduce measurement error, particularly with M-mode imaging. Beginners may also mistake the aorta for the IVC, especially when the vessel appears dilated or pulsatile. Internal jugular vein ultrasound has its own limitations, most notably the lack of a standardized methodology. Approaches described in the literature include assessment of the height of the venous collapse point and changes in vessel diameter or cross-sectional area during respiration or the Valsalva maneuver, among others.

**Figure 4:**
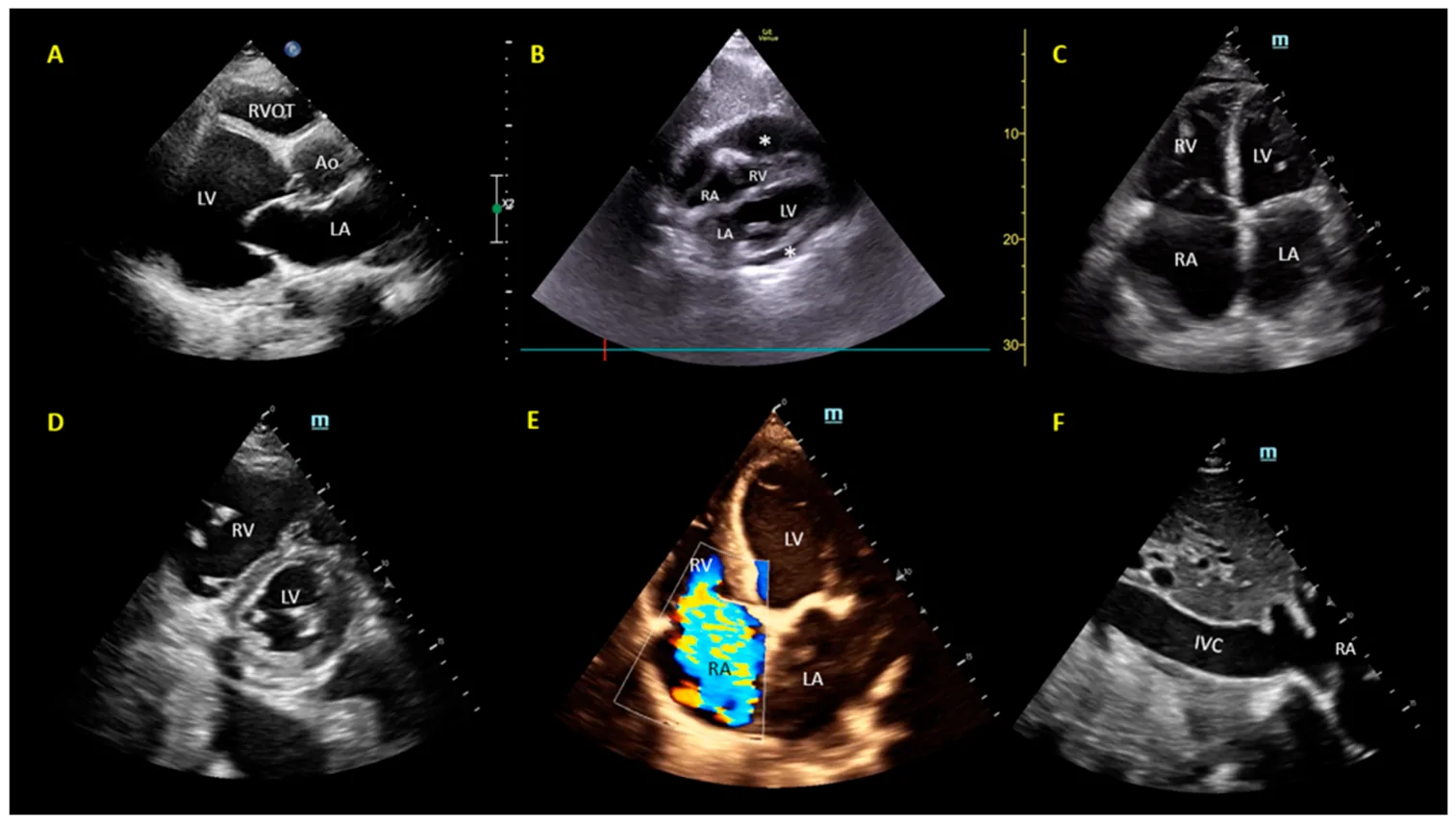
Common abnormalities identified with basic cardiac POCUS. (**A**) Parasternal long-axis view demonstrating a dilated left ventricle suggestive of dilated cardiomyopathy. (**B**) Subxiphoid view showing circumferential pericardial effusion (asterisks). (**C**) Apical four-chamber view demonstrating severe right ventricular (RV) dilation, with the RV larger than the left ventricle (LV), and right atrial (RA) enlargement. (**D**) Parasternal short-axis view showing interventricular septal flattening with a D-shaped LV, consistent with RV pressure or volume overload. (**E**) Apical four-chamber color Doppler demonstrating severe tricuspid regurgitation. (**F**) Dilated inferior vena cava (IVC), suggestive of elevated right atrial pressure.

In addition to grayscale imaging, inspection of valvular structure and color Doppler flow patterns provides important contextual information. For instance, B-lines on lung ultrasound may reflect severe mitral regurgitation, which in turn could be due to conditions such as infective endocarditis rather than isolated fluid overload. In such scenarios, escalating ultrafiltration without concurrent cardiac evaluation may provide only transient symptomatic improvement and, in some cases, may even be harmful by predisposing patients to recurrent decompensation and rehospitalization. Similarly, hypotension in critically ill patients may result from dynamic left ventricular outflow tract obstruction (LVOTO) due to systolic anterior motion of the mitral valve leaflet, particularly in the setting of hypovolemia and left ventricular hypertrophy, the latter being common among patients with kidney disease. In this setting, vasopressors that promote tachycardia may exacerbate LVOTO, whereas heart rate control, reduction in aggravating inotropes, use of vasopressors without β-adrenergic activity, and judicious fluid administration, when appropriate, may improve hemodynamics [14]. Systolic anterior motion can be identified on grayscale imaging by reviewing systolic frames individually or using M-mode. These examples illustrate the importance of integrating focused cardiac ultrasound into nephrology-oriented hemodynamic evaluation rather than interpreting extracardiac findings in isolation. Figure 4 illustrates some common abnormalities identifiable with focused cardiac ultrasound.

Basic cardiac POCUS provides a solid foundation, particularly for clinicians early in their ultrasound training, and can address many common bedside questions. However, nephrologists often evaluate patients later in their clinical course, when hemodynamic questions are less binary and require greater nuance. In these settings, spectral Doppler and quantitative echocardiographic parameters can provide additional information relevant to day-to-day management. These applications are discussed in the following section.

## 3. Advanced Cardiac POCUS

**Figure 5:**
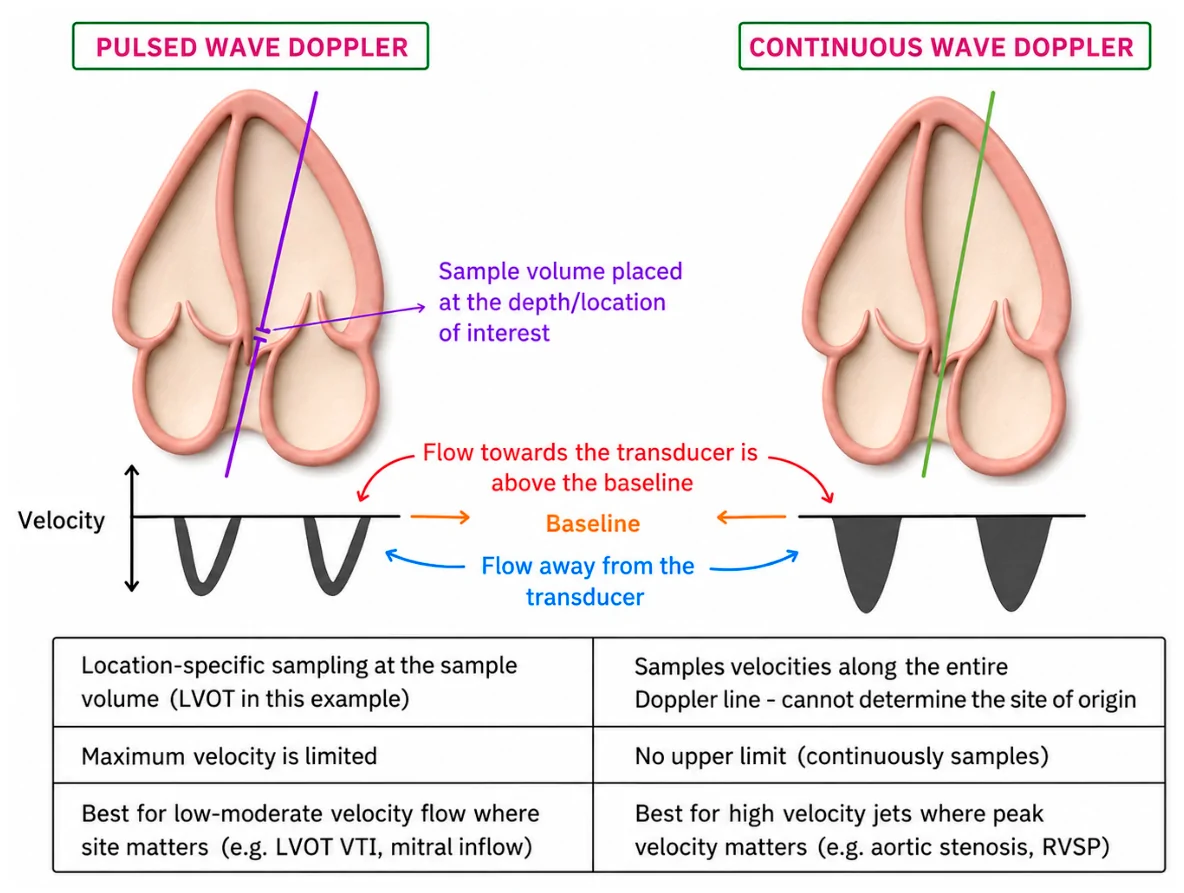
Comparison of pulsed-wave and continuous-wave Doppler. Pulsed-wave Doppler provides location-specific velocity sampling, whereas continuous-wave Doppler records velocities along the entire Doppler beam and permits measurement of higher velocities. The broader, filled spectral envelope typically seen with continuous-wave Doppler reflects the wider range of velocities sampled along the beam.

Advanced cardiac POCUS incorporates spectral Doppler applications, including pulsed-wave Doppler (PWD), continuous-wave Doppler (CWD), and tissue Doppler imaging (TDI). PWD quantifies blood flow velocity at a specific sampling location, whereas CWD measures velocities along the entire Doppler beam and is commonly used to assess high-velocity flows such as transvalvular gradients (Figure 5). TDI, a specialized form of PWD, quantifies myocardial tissue motion and is particularly useful for estimating ventricular relaxation and diastolic function. These applications require substantially greater technical proficiency than basic cardiac POCUS. Accurate Doppler measurements depend heavily on appropriate alignment between the ultrasound beam and the direction of blood or tissue motion. Measurements obtained from foreshortened, tilted, or otherwise suboptimal cardiac views may therefore lead to significant inaccuracies and potentially inappropriate clinical decisions. In addition, many Doppler-derived parameters rely on physiologic assumptions and have important limitations that may not apply uniformly across all clinical settings. Proper interpretation therefore requires familiarity not only with image acquisition, but also with the underlying hemodynamic principles, waveform quality, respiratory and cardiac cycle variation, and the specific contexts in which a given parameter or formula is valid. POCUS informs a hemodynamic hypothesis; it does not independently point to therapy. Technically inadequate or clinically discordant findings should prompt comprehensive echocardiography or specialist assessment.

Below, we discuss some of the commonly used spectral Doppler applications relevant to nephrology practice.

### 3.1. Stroke Volume Assessment

Stroke volume assessment is particularly relevant to nephrologists, as it can help distinguish low-output states potentially related to volume depletion from euvolemic and high-output states, thereby informing therapeutic decisions regarding intravenous fluids, diuretics, and vasoactive agents. This distinction is important because the IVC may appear small and collapsible across these conditions, reflecting normal RAP rather than intravascular volume status. In patients with preserved or hyperdynamic LV systolic function and non-elevated RAP, reduced stroke volume may raise suspicion for hypovolemia, whereas preserved or elevated stroke volume may suggest adequate or increased forward flow, as seen in hyperdynamic states such as cirrhosis. Importantly, low stroke volume alone does not establish volume depletion or predict fluid responsiveness and must be interpreted in the broader clinical and hemodynamic context. This integrated assessment may be particularly useful in patients with cirrhosis presenting with AKI or hyponatremia when considering fluid administration, as well as in dialysis patients with high-flow arteriovenous fistulas and suspected high-output cardiac failure [15,16].

Conversely, a low stroke volume should prompt consideration of several potential mechanisms beyond hypovolemia. In severe mitral regurgitation, forward stroke volume may be reduced because a substantial proportion of left ventricular ejection is directed back into the left atrium, underscoring the importance of color Doppler assessment when the cause of low forward flow is unclear. Reduced forward flow may also occur in heart failure with reduced ejection fraction, although contemporary data suggest that venous congestion may exert a greater influence on renal dysfunction than impaired forward flow [17]. Similarly, marked RV pressure or volume overload may impair left ventricular filling through ventricular interdependence, thereby reducing stroke volume. In such cases, however, the IVC is expected to be plethoric.

Serial stroke volume assessment may also be useful during aggressive diuresis or ultrafiltration, as compensatory tachycardia may preserve cardiac output, while increased vascular resistance may maintain blood pressure despite a clinically meaningful decline in stroke volume. Trending stroke volume may therefore help detect excessive reductions in forward flow during decongestion or evolving cardiogenic shock. In the latter setting, POCUS findings should be integrated with clinical markers of tissue perfusion, such as capillary refill time, to determine whether reduced flow is associated with impaired end-organ perfusion [18].

**Figure 6:**
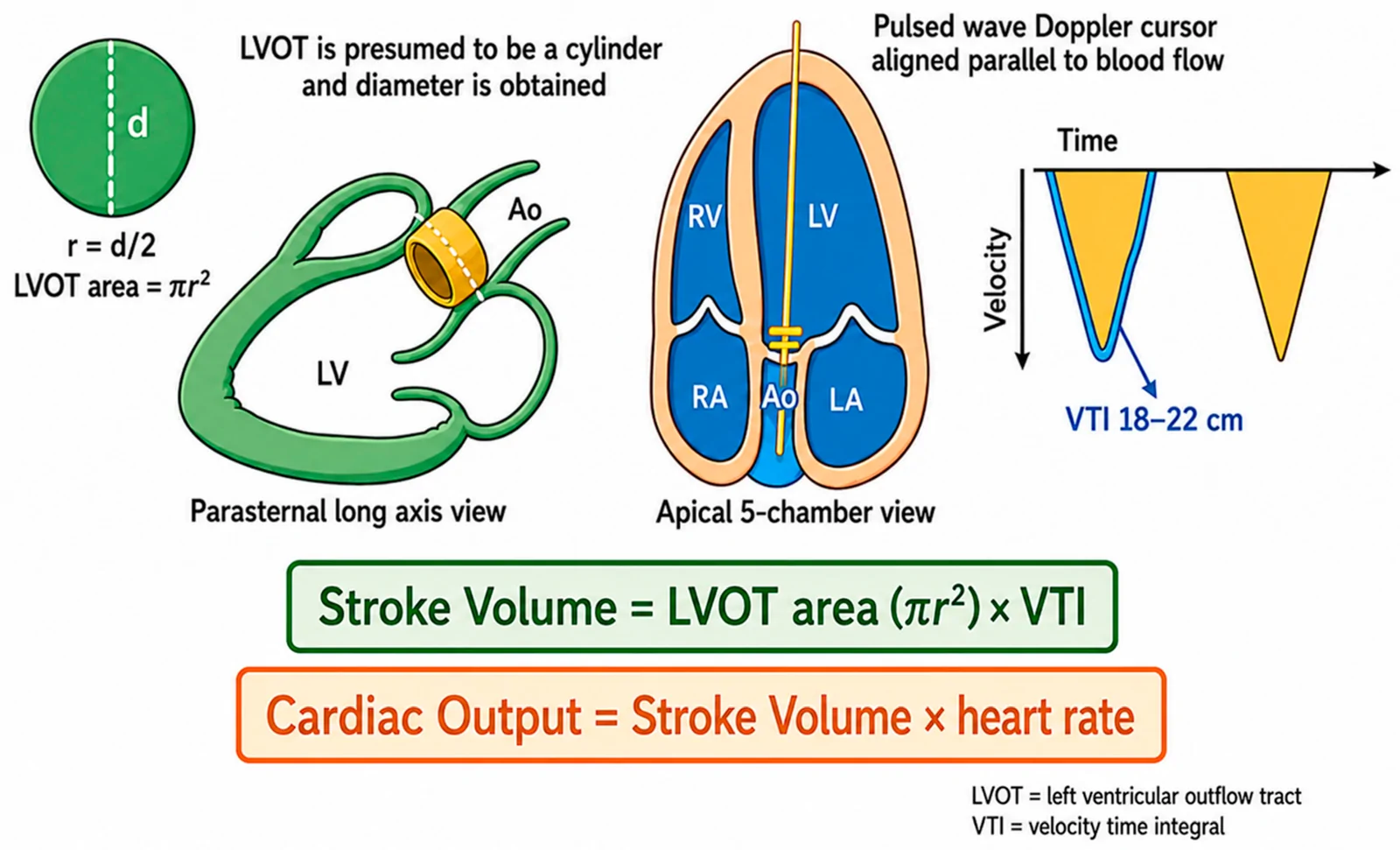
Doppler estimation of stroke volume and cardiac output. Stroke volume is calculated from the left ventricular outflow tract (LVOT) cross-sectional area and velocity-time integral (VTI), with cardiac output derived from stroke volume and heart rate. Inadequate alignment of the Doppler beam with blood flow commonly underestimates VTI, while errors in LVOT diameter are amplified because the measurement is squared when calculating cross-sectional area. VTI may also be overestimated if the sample volume is positioned within the flow-acceleration zone in aortic stenosis. Careful acquisition technique is therefore essential for reliable measurements.

**Figure 7:**
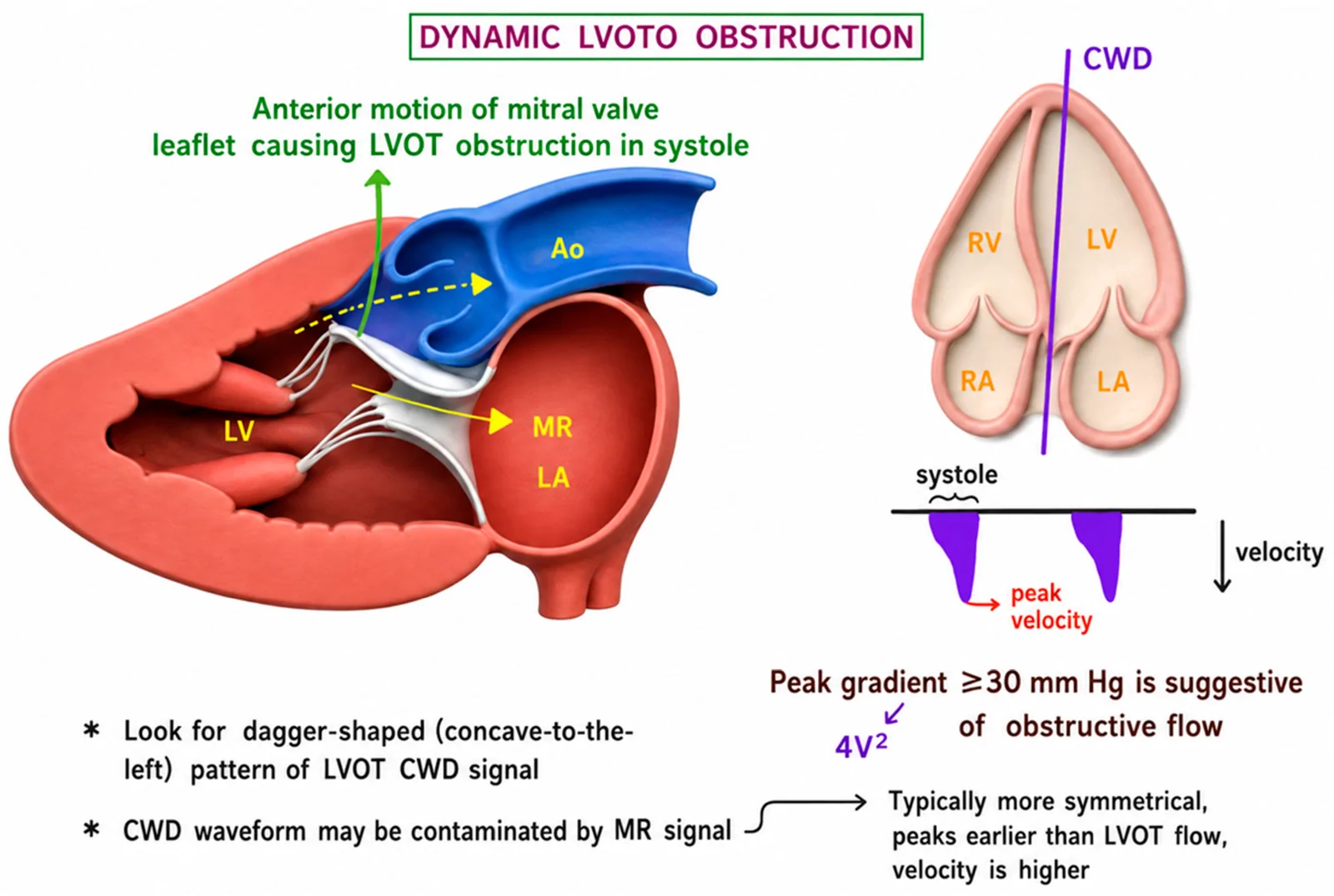
Dynamic left ventricular outflow tract obstruction (LVOTO). Systolic anterior motion of the mitral valve leaflet produces dynamic LVOT obstruction and may be accompanied by mitral regurgitation (MR). Continuous-wave Doppler (CWD) across the LVOT demonstrates a characteristic late-peaking, dagger-shaped systolic envelope, reflecting progressive narrowing of the LVOT during systole, with a peak gradient ≥ 30 mm Hg supporting LVOTO. Care should be taken to distinguish the LVOT signal from the typically higher-velocity MR signal, which may contaminate the Doppler envelope.

Sonographically, stroke volume can be estimated from the LVOT velocity-time integral (VTI) obtained using PWD and the LVOT cross-sectional area. When the LVOT diameter is not measured, VTI alone can serve as a practical surrogate for monitoring changes in stroke volume, particularly when assessing response to hemodynamic interventions, as LVOT diameter remains constant within an individual (Figure 6). LVOT VTI values of approximately 18–22 cm are generally considered normal [19]. In hospitalized patients, broader LVOT VTI thresholds of <15 cm or >25 cm may serve as practical, expert-derived reference points for considering low or high forward flow, respectively, while accounting for physiologic variability and minor differences in Doppler alignment. For example, a VTI of 23–24 cm alone does not establish a high-output state, whereas values of 16–17 cm may reflect a higher heart rate with preserved cardiac output. When dynamic LVOTO is suspected, CWD interrogation across the LVOT is useful and typically demonstrates a late-peaking, dagger-shaped systolic waveform. A peak LVOT gradient ≥ 30 mmHg, calculated as 4 × (peak velocity)^2^, is consistent with dynamic LVOTO [20] (Figure 7).

### 3.2. Left Ventricular Filling Pressures

The terms “left ventricular filling pressure (LVFP)” and “left atrial pressure (LAP)” are often used interchangeably in echocardiography, as noninvasive estimation of LVFP primarily reflects mean LAP and its invasive surrogate, mean pulmonary capillary wedge pressure, rather than left ventricular end-diastolic pressure. Assessment of LVFP is particularly useful when cardiogenic and primary pulmonary causes of increased extravascular lung water are difficult to distinguish, given the nonspecific nature of pulmonary B-lines. This distinction has important implications for fluid management, as continued diuresis may compromise renal perfusion without substantially improving lung water when increased pulmonary permeability, rather than elevated hydrostatic pressure, is the predominant mechanism. Conversely, LVFP assessment may help guide fluid administration in patients at high risk for pulmonary edema, such as those with cirrhosis and AKI, allowing a more judicious approach to volume expansion. LVFP assessment may also be useful in the outpatient setting to optimize diuretic therapy before symptoms develop, particularly when Doppler findings suggest rising filling pressures compared with prior examinations.

**Figure 8:**
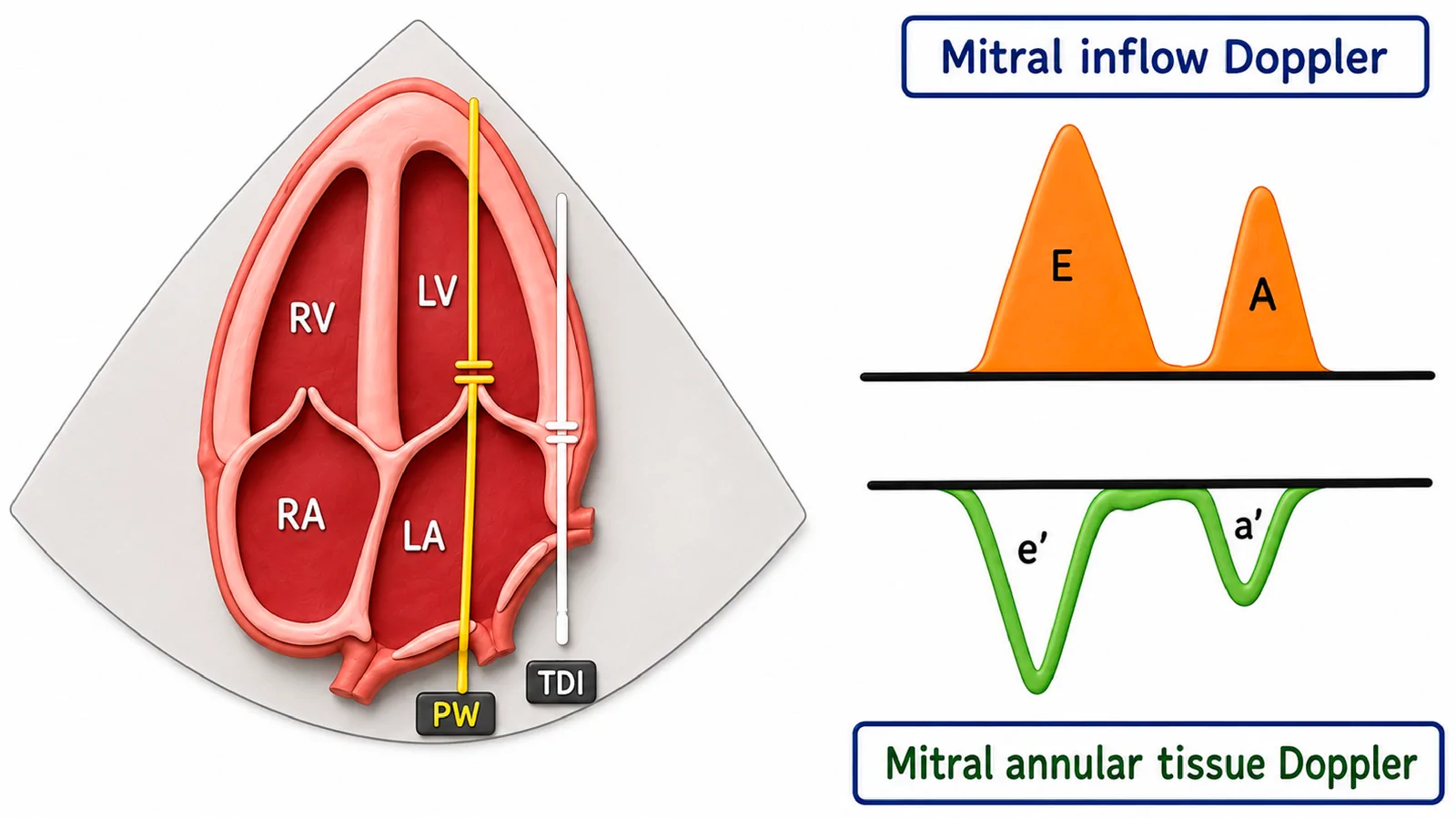
Mitral inflow and mitral annular tissue Doppler. Pulsed wave (PW) Doppler demonstrates the transmitral early (E) and late (A) diastolic filling waves, while tissue Doppler imaging (TDI) measures the corresponding early (e′) and late (a′) mitral annular velocities. An E/A ratio ≥ 2 indicates a restrictive filling pattern and, in patients with reduced left ventricular ejection fraction, may be sufficient to suggest elevated left atrial pressure (LAP). Otherwise, E/e′ can be used to assess LAP as described in the text. Importantly, the E-wave is preload dependent and may be increased in volume overload or mitral regurgitation, with the velocity potentially decreasing following volume removal.

Sonographic assessment of LAP begins with TDI of the mitral annulus to measure the early diastolic (e′) velocity. Septal e′ ≤ 6 cm/s, lateral e′ ≤ 7 cm/s, or average e′ ≤ 6.5 cm/s suggests impaired myocardial relaxation. Mitral inflow is subsequently assessed using PWD to obtain early (E) and late atrial (A) diastolic velocities and the E/A ratio, followed by calculation of E/e′ (Figure 8). An E/e′ ≥ 15 using septal e′, ≥ 13 using lateral e′, or ≥14 using average e′ supports elevated LAP [21]. In patients with reduced LV ejection fraction, an E/A ratio ≥ 2 supports a restrictive filling pattern and elevated LAP but should be interpreted alongside the other diastolic indices. Mean pulmonary capillary wedge pressure can also be estimated using the formula [1.24 × (lateral E/e′) + 1.9 mmHg], with E/e′ + 4 serving as a convenient bedside approximation; however, these estimates should not be interpreted as precise equivalents of invasively measured filling pressures [22]. Furthermore, E/e′ should not be interpreted in isolation, as its relationship with filling pressure and ability to track changes vary with ventricular function, mitral valve disease, rhythm, and loading conditions [23]. Additional cardiac POCUS findings can provide complementary information. A peak TR velocity ≥ 2.8 m/s measured with CWD suggests elevated pulmonary artery systolic pressure and, in the absence of intrinsic pulmonary or pulmonary vascular disease, may support elevated LAP due to passive transmission of left-sided filling pressures [21]. Qualitative left atrial enlargement on the parasternal long-axis view may provide an additional clue to chronic diastolic dysfunction but should not be interpreted as evidence of acutely elevated LAP. Although left atrial volume index and reservoir strain provide more objective measures of left atrial remodeling and function, respectively, these measurements are not routinely incorporated into POCUS because of time and technical constraints.

### 3.3. Right-Sided Pressures

**Figure 9:**
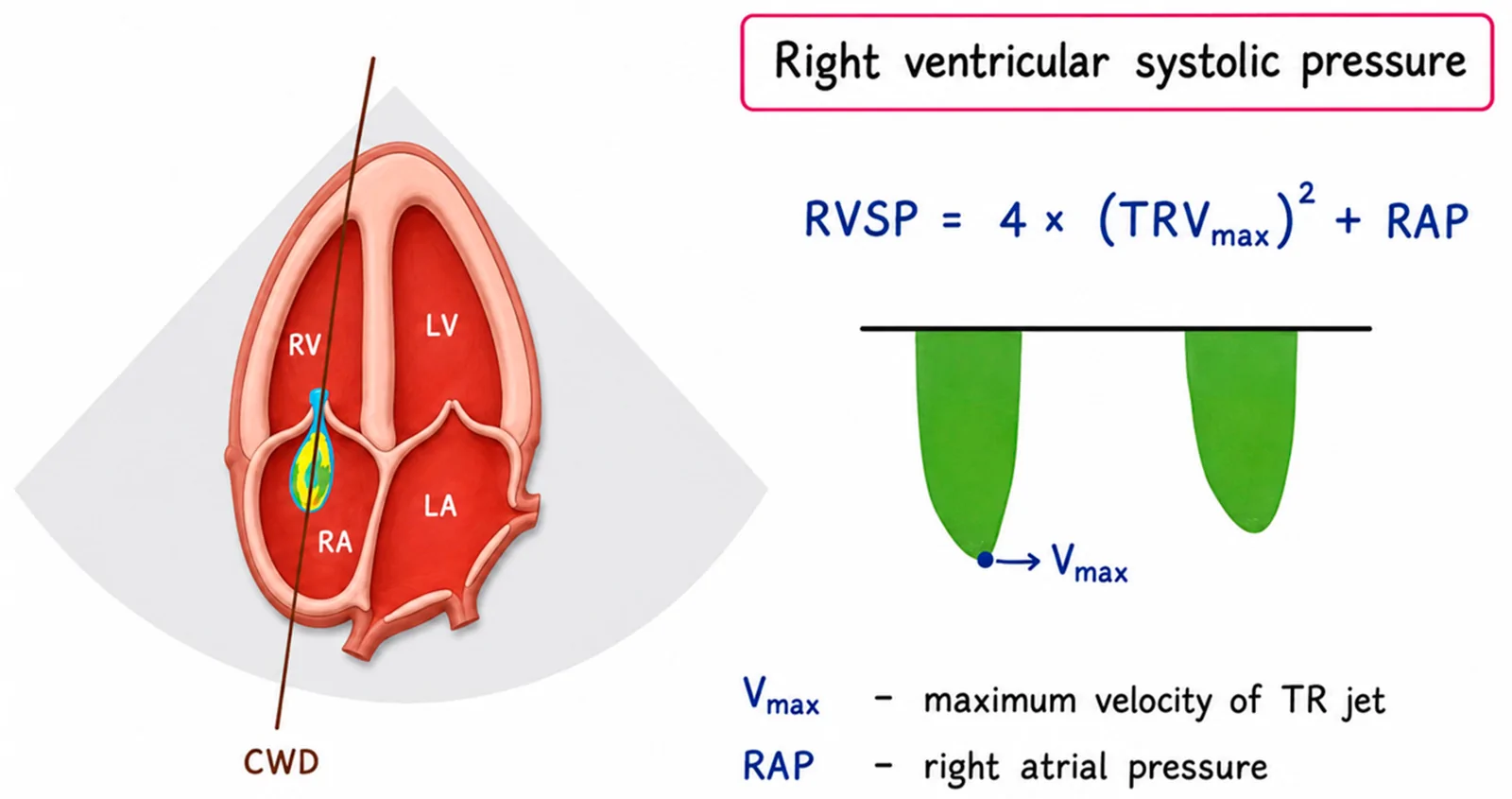
Doppler estimation of right ventricular systolic pressure (RVSP). Continuous-wave Doppler (CWD) is aligned with the tricuspid regurgitation (TR) jet to obtain its peak velocity (TRVmax). The RV–right atrial systolic pressure gradient is calculated using the simplified Bernoulli equation (4 × TRVmax^2^), and estimated right atrial pressure (RAP) is added to derive RVSP. RAP can be estimated using inferior vena cava ultrasound or obtained directly from central venous pressure when a catheter is present.

RAP reflects RV filling pressure and is determined by the interaction of venous return, RV compliance, TR, and pericardial constraint. Pulmonary arterial or RV systolic pressure (RVSP) influences RAP indirectly through its effects on RV remodeling and function. When RV pressure overload is suspected, RVSP can be estimated from the peak TR velocity obtained by continuous-wave Doppler: RVSP = 4 × (TR velocity)^2^ + estimated RAP (Figure 9). In the absence of RV outflow or pulmonic valve obstruction, RVSP approximates pulmonary artery systolic pressure (PASP). Values ≥ 35 mmHg are generally abnormal, while >60 mmHg indicates marked elevation [24,25]. Of note, severe tricuspid regurgitation may lead to underestimation of RVSP due to rapid RV–RA pressure equalization. Also, because RAP is estimated from IVC parameters using relatively broad ranges, some guidelines favor TR velocity over calculated RVSP when assessing the probability of pulmonary hypertension.

The clinical significance of an elevated RVSP depends on its mechanism and trajectory. Potential causes include elevated LVFP, pulmonary parenchymal or vascular disease, and acute RV afterload elevation from pulmonary embolism. Accordingly, RVSP may decrease following decongestion, pulmonary embolism treatment, or reduction in pulmonary vascular resistance, whereas chronically elevated values may change little in established pulmonary hypertension [24]. Comparison with prior outpatient measurements can therefore be informative in hospitalized patients with known pulmonary hypertension.

**Figure 10:**
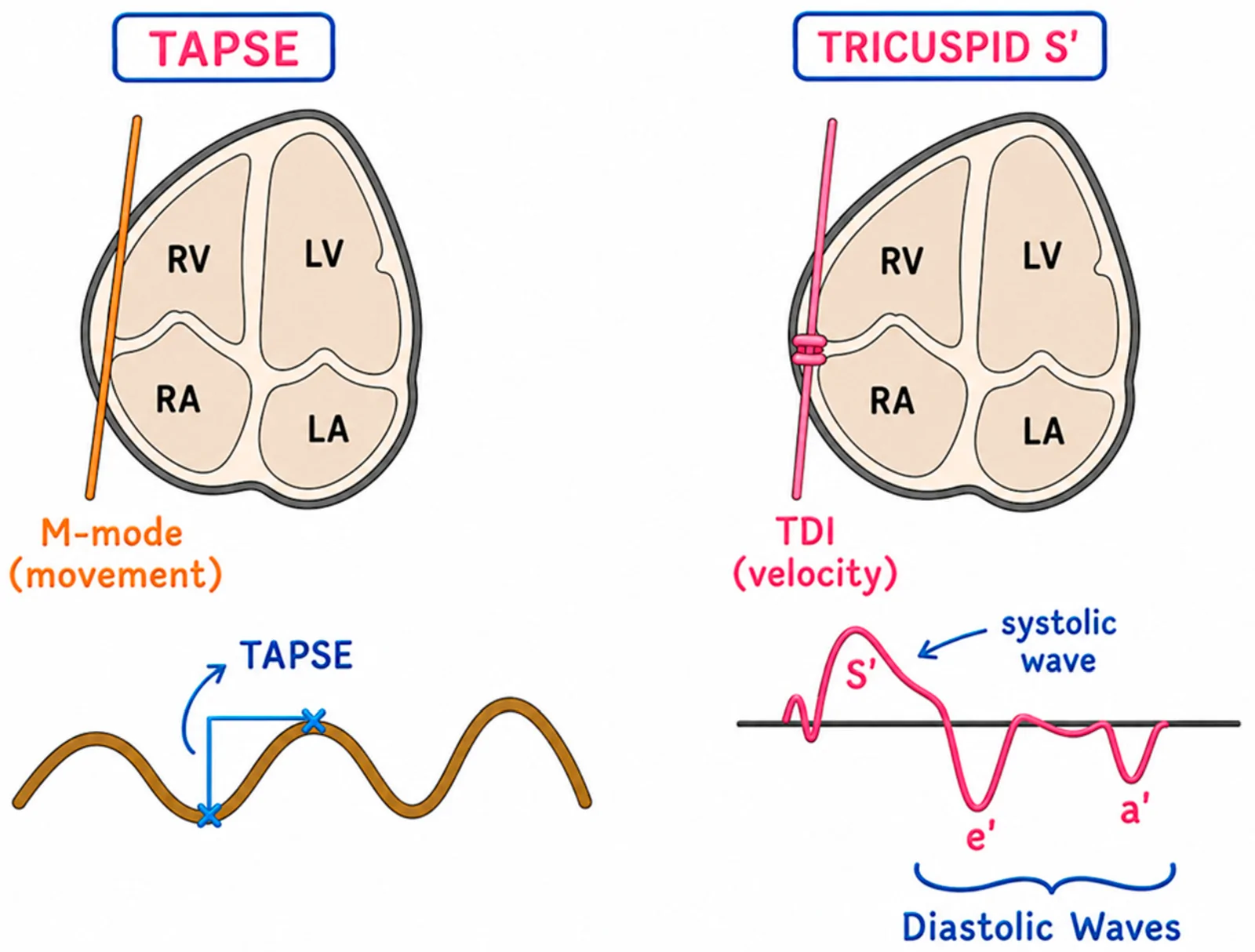
Cardiac ultrasound assessment of right ventricular systolic function. Tricuspid annular plane systolic excursion (TAPSE) measures longitudinal systolic motion of the lateral tricuspid annulus using M-mode, with >17 mm considered normal. Tricuspid annular S′ measures systolic tissue velocity using tissue Doppler imaging (TDI), with ≥9.5–10 cm/s considered normal. Both parameters assess primarily basal lateral longitudinal RV function and are angle dependent; alignment of the lateral tricuspid annulus with the ultrasound beam is essential to avoid underestimation.

Integrating RVSP with other POCUS findings can help characterize the predominant pulmonary hypertension phenotype, although echocardiography alone cannot definitively establish pre- versus post-capillary physiology. These patterns should be regarded as physiologic clues rather than a noninvasive substitute for hemodynamic classification, which may require right-heart catheterization when clinically consequential. Elevated RVSP accompanied by left atrial enlargement, LV hypertrophy or dysfunction, and elevated E/e′ favors a post-capillary pattern. Conversely, disproportionate right-sided pressure overload with RV dilation or systolic dysfunction, in the absence of elevated LVFP, suggests a pre-capillary pattern [25]. RV systolic function can be assessed by TAPSE on grayscale or M-mode imaging or by TDI-derived S′ velocity (Figure 10). These patterns can help identify the likely contributors to pulmonary pressure elevation, inform assessment of congestion and RV function, and prioritize further evaluation [24,25]. In a patient with worsening renal function and venous congestion, integration of these findings with the clinical assessment may support individualized decongestion and monitoring of its hemodynamic effects. Disproportionate right-sided pressure overload without evidence of elevated LVFP should prompt evaluation for pulmonary vascular or parenchymal disease and, when appropriate, specialist referral. Disease-specific treatment requires confirmation of the underlying diagnosis, including invasive hemodynamic assessment when indicated, rather than selection on the basis of the echocardiographic pattern alone. When RV function is impaired, decongestion requires particular attention to maintaining adequate forward flow and organ perfusion [24].

**Figure 11:**
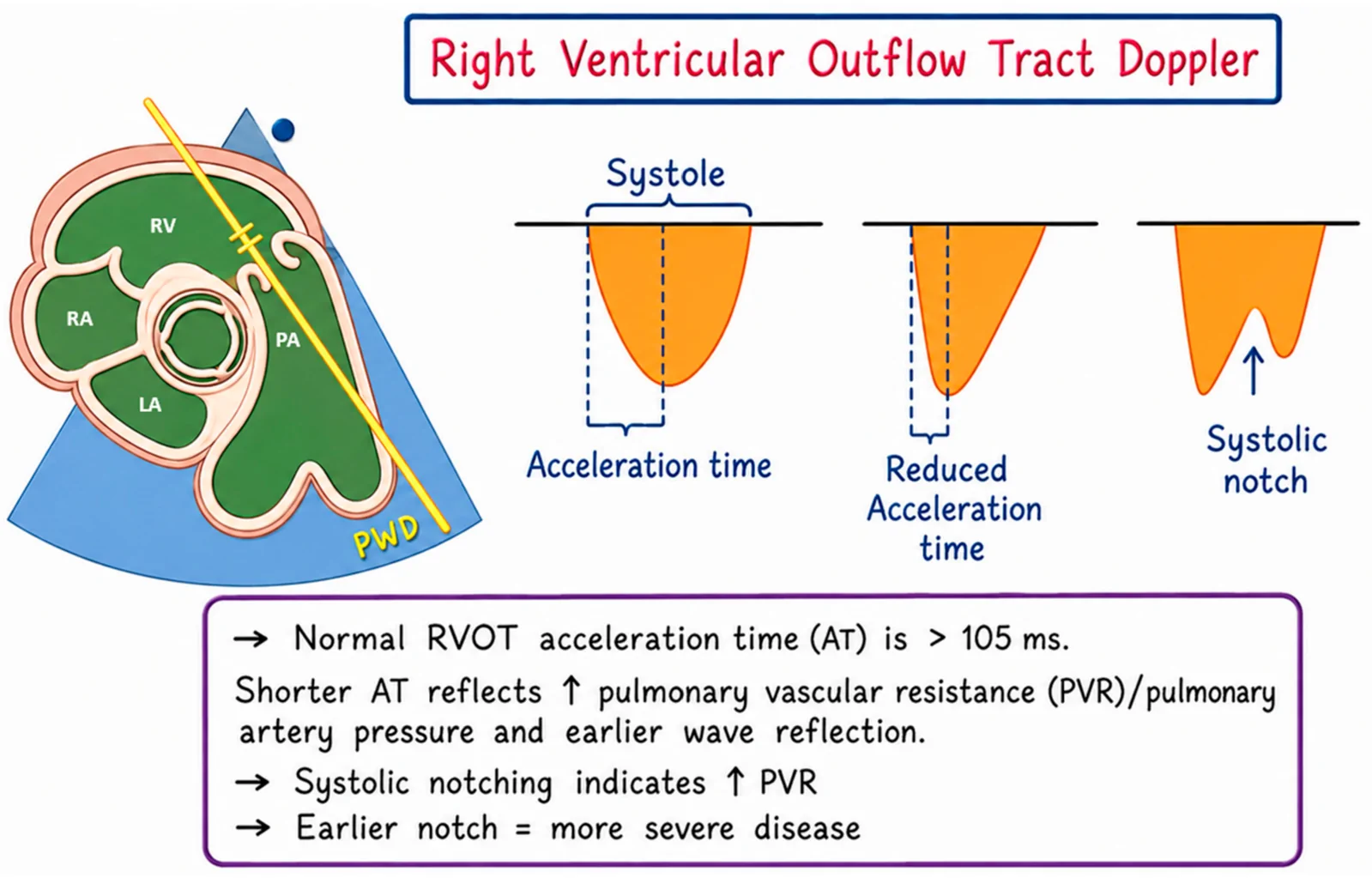
Right ventricular outflow tract (RVOT) pulsed-wave Doppler. RVOT acceleration time is measured from the onset of systolic flow to peak velocity and is normally >105 ms. Shortening of acceleration time and development of systolic notching suggest increasing pulmonary vascular load, with earlier notching generally reflecting more severe disease.

**Figure 12:**
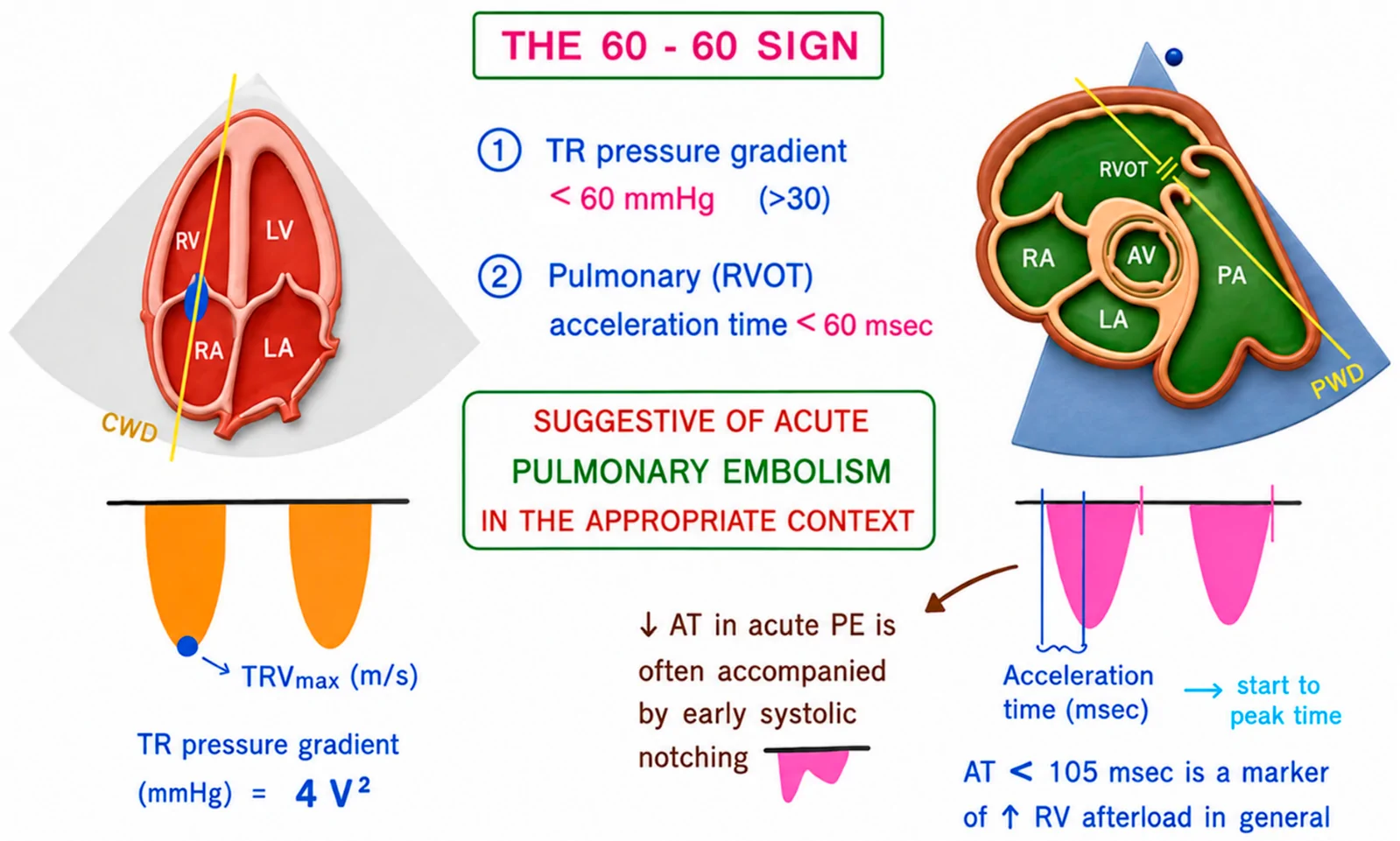
The 60/60 sign in acute pulmonary embolism. The 60/60 sign combines an RVOT acceleration time < 60 ms with a tricuspid regurgitation systolic pressure gradient < 60 mmHg (but typically >30 mmHg) and, in the appropriate clinical context, supports acute pulmonary embolism. Shortened acceleration time (AT) is often accompanied by early systolic notching of the RVOT Doppler waveform.

RV outflow tract (RVOT) PWD provides additional information about pulmonary vascular load. Systolic notching suggests increased pulmonary vascular resistance, whereas a smooth, rounded systolic envelope is more consistent with lower resistance. In the appropriate clinical setting, an early systolic notch producing a spike-and-dome configuration with a shortened acceleration time (<60–80 ms) should raise suspicion for hemodynamically significant pulmonary embolism [26] (Figure 11). A related finding, the 60/60 sign, combines an RVOT acceleration time < 60 ms with a TR systolic pressure gradient < 60 mmHg (typically >30 mmHg) and is relatively specific, although insensitive, for acute pulmonary embolism [27,28] (Figure 12). This pattern reflects abrupt RV afterload elevation with rapid deceleration of pulmonary flow, while the acutely stressed, nonhypertrophied RV is unable to generate the markedly elevated pressures characteristic of chronic pulmonary hypertension. Recognition of these findings is particularly relevant to nephrologists given the increased risk of pulmonary embolism in patients with kidney disease, especially those receiving dialysis or with nephrotic syndrome [29].

**Figure 13:**
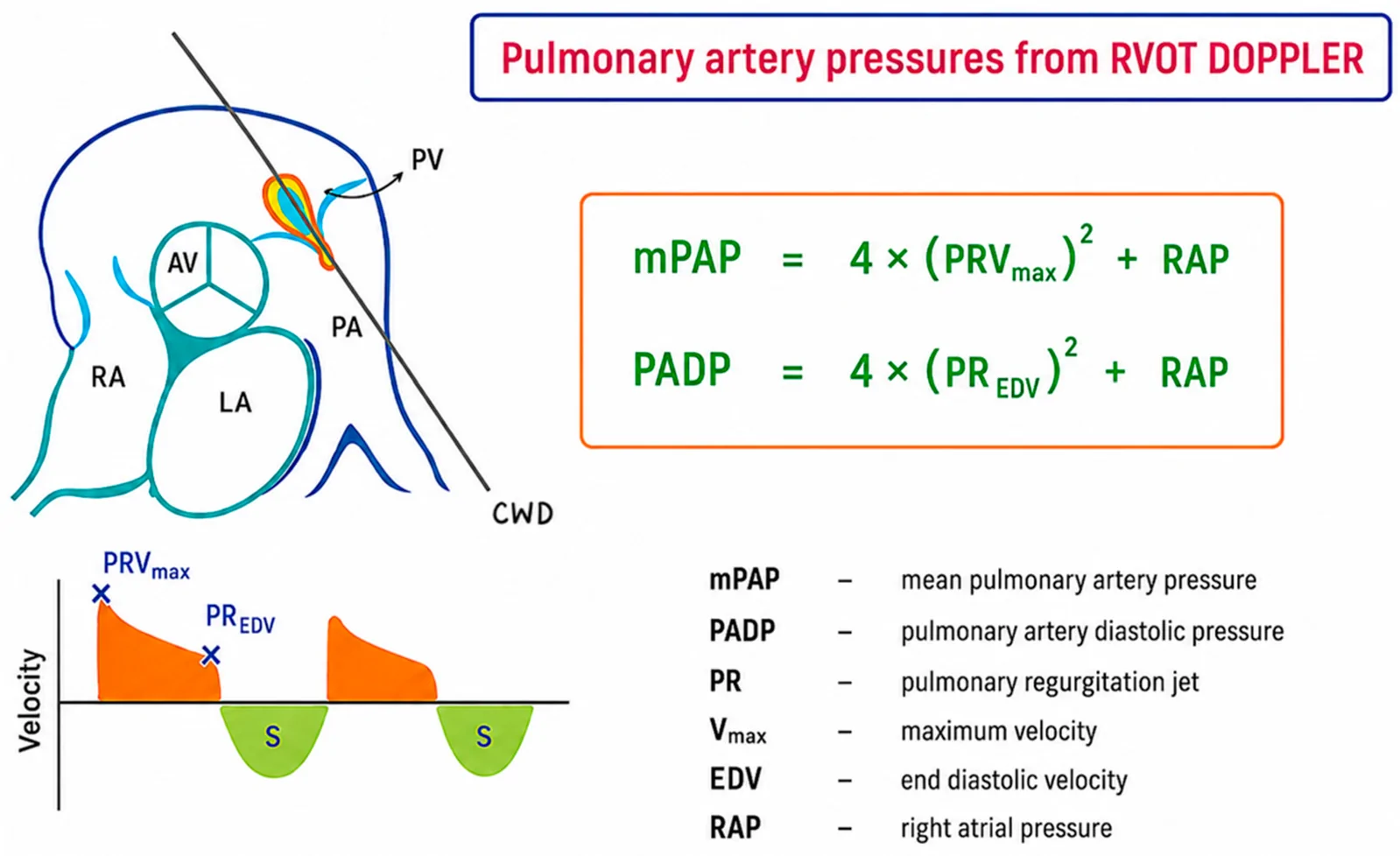
Estimation of pulmonary artery pressures using pulmonary regurgitation Doppler. Continuous-wave Doppler (CWD) through the pulmonary valve records the pulmonary regurgitation (PR) jet. Mean pulmonary artery pressure (mPAP) is estimated from the peak PR velocity (PRVmax), whereas pulmonary artery diastolic pressure (PADP) is estimated from the end-diastolic PR velocity (PR_EDV_), using the simplified Bernoulli equation and adding estimated right atrial pressure (RAP).

When a pulmonary regurgitation (PR) jet is present, CWD interrogation can be used to estimate both mean pulmonary artery pressure (mPAP) and pulmonary artery diastolic pressure (PADP) from the parasternal short axis view. mPAP is estimated as 4 × (early PR velocity)^2^ + RAP, whereas PADP is calculated from the end-diastolic PR velocity as 4 × (end-diastolic PR velocity)^2^ + RAP [30] (Figure 13). When pulmonary vascular resistance is not elevated, PADP may approximate pulmonary capillary wedge pressure and provide complementary information about left-sided filling pressures. This relationship becomes less reliable in pulmonary vascular disease, and the Doppler-derived estimate is additionally influenced by PR signal quality and the accuracy of estimated RAP. Interpreted alongside other echocardiographic findings and clinical evidence of congestion, PADP may contribute to assessment and reassessment during diuretic therapy, without serving as an isolated treatment target.

### 3.4. VExUS

**Figure 14:**
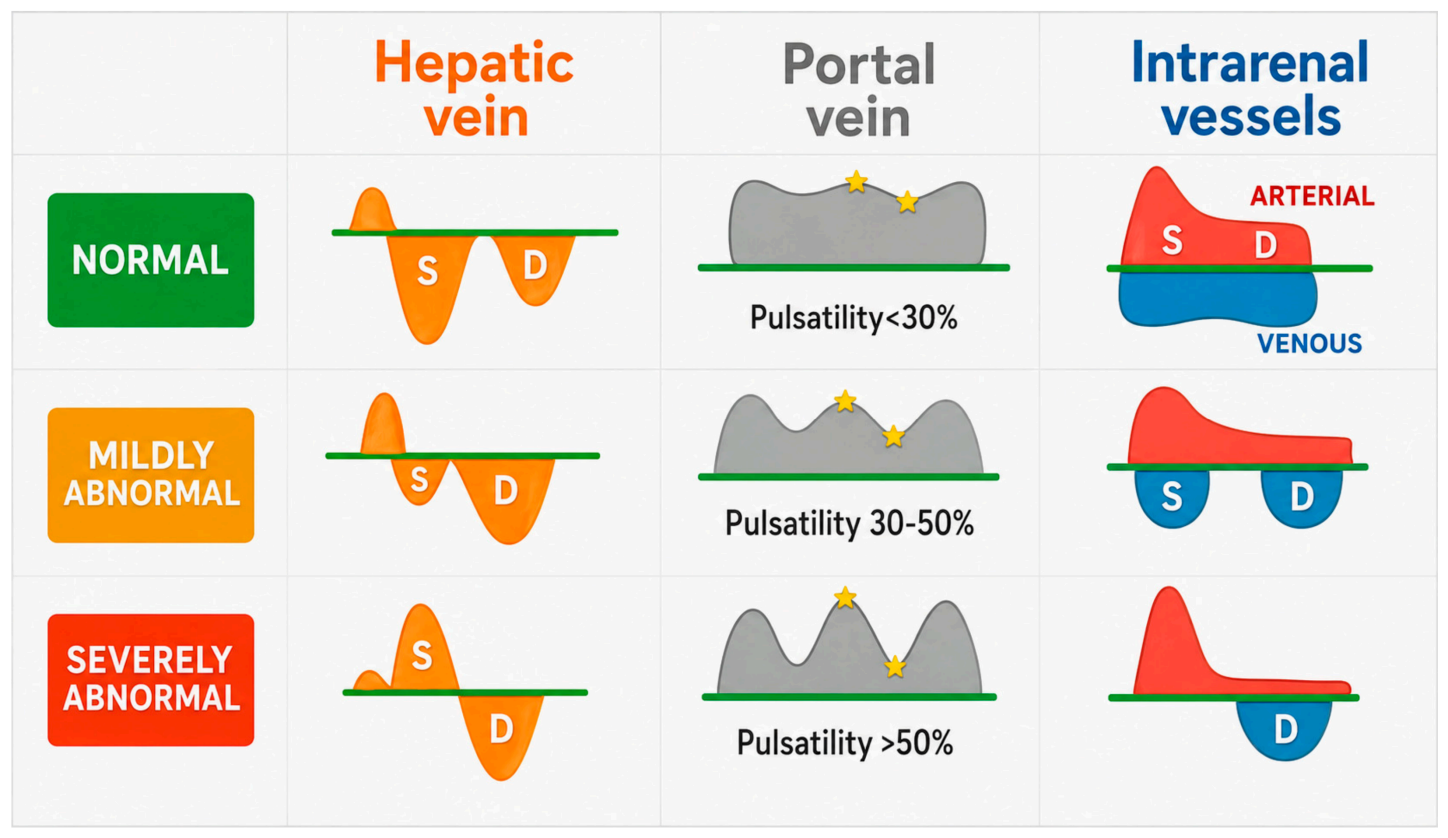
Normal and abnormal venous Doppler waveforms used in VExUS assessment. In the hepatic vein, a normal pattern is systolic dominant (S > D), mild abnormality is characterized by S < D, and severe abnormality by systolic flow reversal; subsequent modifications have also considered absent systolic flow as severely abnormal without prominent reversal. Portal vein pulsatility is <30% normally, 30–50% with mild abnormality, and >50% with or without flow reversal when severely abnormal. Yellow asterisks on the waveform represent measurement points to calculate pulsatility (same cardiac cycle). Intrarenal venous flow is normally relatively continuous; a biphasic pattern with distinct systolic and diastolic components is mildly abnormal, whereas isolated diastolic flow is severely abnormal. Hepatic vein interpretation is facilitated by simultaneous ECG, while the accompanying intrarenal arterial waveform can serve as a built-in cardiac timing reference.

**Figure 15:**
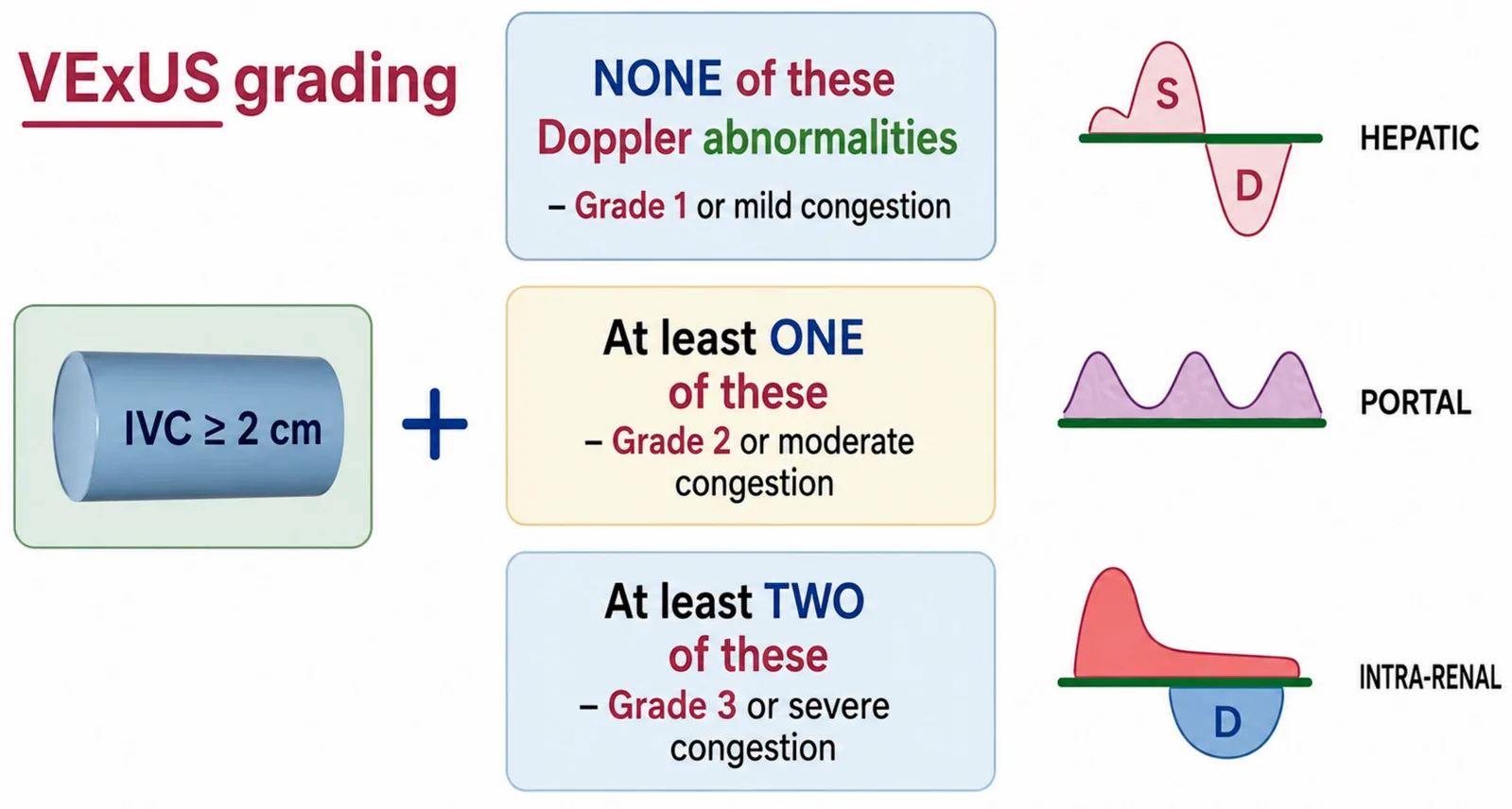
VExUS grading of systemic venous congestion. In the presence of a dilated IVC (≥2 cm), absence of severe Doppler abnormalities corresponds to grade 1 (mild) congestion, one severely abnormal waveform to grade 2 (moderate), and two or more to grade 3 (severe) congestion. The right panel illustrates the severely abnormal hepatic, portal, and intrarenal venous Doppler patterns. In the absence of IVC dilation, venous congestion is classified as grade 0.

VExUS quantifies systemic venous congestion using IVC ultrasound combined with hepatic, portal, and intrarenal venous Doppler. Figure 14 and Figure 15 illustrate normal and abnormal waveforms and the corresponding scoring system. Although VExUS is not formally part of focused cardiac ultrasound, it represents a valuable extension of hemodynamic assessment, particularly for nephrologists evaluating systemic venous congestion and its renal consequences. It can also be used to monitor response to therapy, as venous Doppler waveforms change with decongestion. By assessing the downstream effects of elevated RAP, VExUS provides information beyond pressure estimation alone. IVC ultrasound asks whether RAP is elevated; VExUS asks whether that pressure is being transmitted to the systemic venous circulation. In the original study of cardiac surgery patients, VExUS outperformed RAP in predicting subsequent AKI, likely because venous Doppler abnormalities reflect not only RAP but also venous compliance and the transmission of pressure through the venous circulation [2]. Subsequent studies across diverse clinical settings have demonstrated emerging prognostic value in patients with heart failure and kidney dysfunction [31,32]. Serial improvement in VExUS, particularly when integrated with lung ultrasound, has also been associated with favorable clinical outcomes [33]. These associations do not establish benefit from VExUS-guided treatment. Although current data suggest improved decongestion, evidence for improved kidney recovery or other patient-centered outcomes remains limited. Also, it is important to recognize that VExUS, like IVC ultrasound, is not a specific marker of intravascular volume and should not be used interchangeably with volume status assessment. Accordingly, the same physiologic and pathologic factors that influence the IVC can also affect VExUS interpretation. Readers interested in VExUS grading, its extensions, acquisition techniques, and common technical pitfalls are referred to our previous publications [3,34,35].

## 4. Conclusions

Knowledge of cardiac ultrasound findings is valuable in nephrology practice, particularly when nephrologists can acquire and interpret images at the bedside to address specific clinical questions. Unlike a comprehensive echocardiographic examination, POCUS allows clinicians to selectively assess parameters relevant to an immediate hemodynamic problem and repeat these assessments as frequently as needed to evaluate response to therapy. Cardiac findings can also be integrated with lung, vascular, and abdominal POCUS within the same examination, providing a more cohesive assessment while potentially reducing the need for multiple formal studies, patient discomfort, and fragmentation of care. However, appropriate training remains essential for accurate image acquisition and interpretation. Although professional society-led training efforts have recently begun to emerge, substantial work remains to establish broader competency and standardized education. The authors therefore do not advocate performing the sonographic applications discussed here, particularly advanced Doppler techniques, without appropriate formal training and demonstration of competency.

Current evidence does not establish that POCUS itself improves patient-centered outcomes; however, this expectation should be considered in the context of its role as a diagnostic modality. Improved diagnostic accuracy can translate into better outcomes only when the information obtained informs an effective therapeutic intervention that itself favorably affects those outcomes. For example, even precise characterization of hemodynamics in a patient with cirrhosis and AKI cannot overcome the prognosis of advanced liver disease when definitive therapy requires liver transplantation. Nevertheless, identifying clinically meaningful abnormalities and avoiding potentially harmful interventions, such as unnecessary fluid administration leading to pulmonary edema, represent potential clinical benefits even in the absence of demonstrated mortality reduction. Importantly, POCUS has consistently demonstrated greater diagnostic accuracy than conventional physical examination for several clinically relevant findings, supporting its use as an extension of bedside assessment rather than requiring mortality benefit to justify every application.

Future research should test whether integrating cardiac and multisystem POCUS into predefined management pathways changes treatment decisions and improves patient-centered outcomes in specific nephrology-related conditions. Artificial intelligence tools that facilitate image acquisition, interpretation, and workflow are promising, particularly for less experienced users. However, current technologies do not replace the clinical reasoning required to synthesize sonographic findings with the broader clinical picture and translate them into appropriate management decisions. Ultimately, the value of POCUS in nephrology lies not merely in acquiring more information, but in enabling the treating clinician to obtain the right information at the right time, interpret it in context, and use it to guide patient care.

## Data Availability

No new data were generated or analyzed in this review. Data sharing is not applicable to this article.
